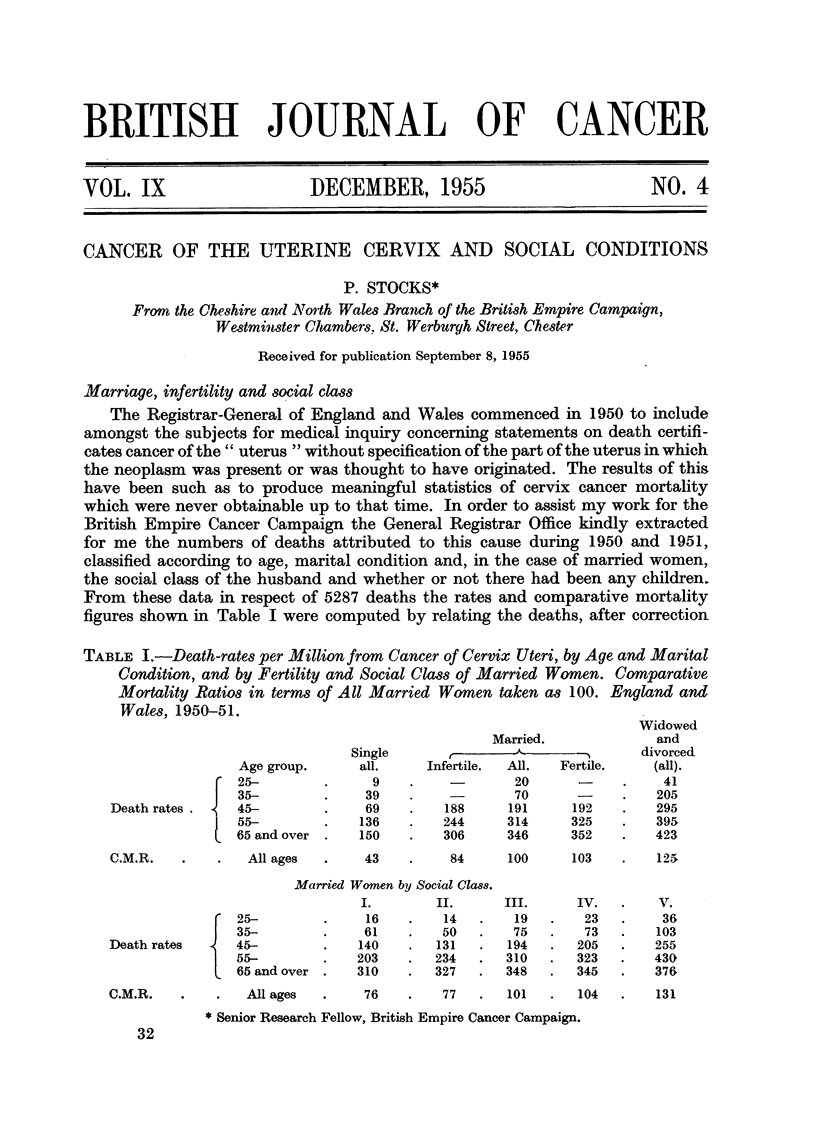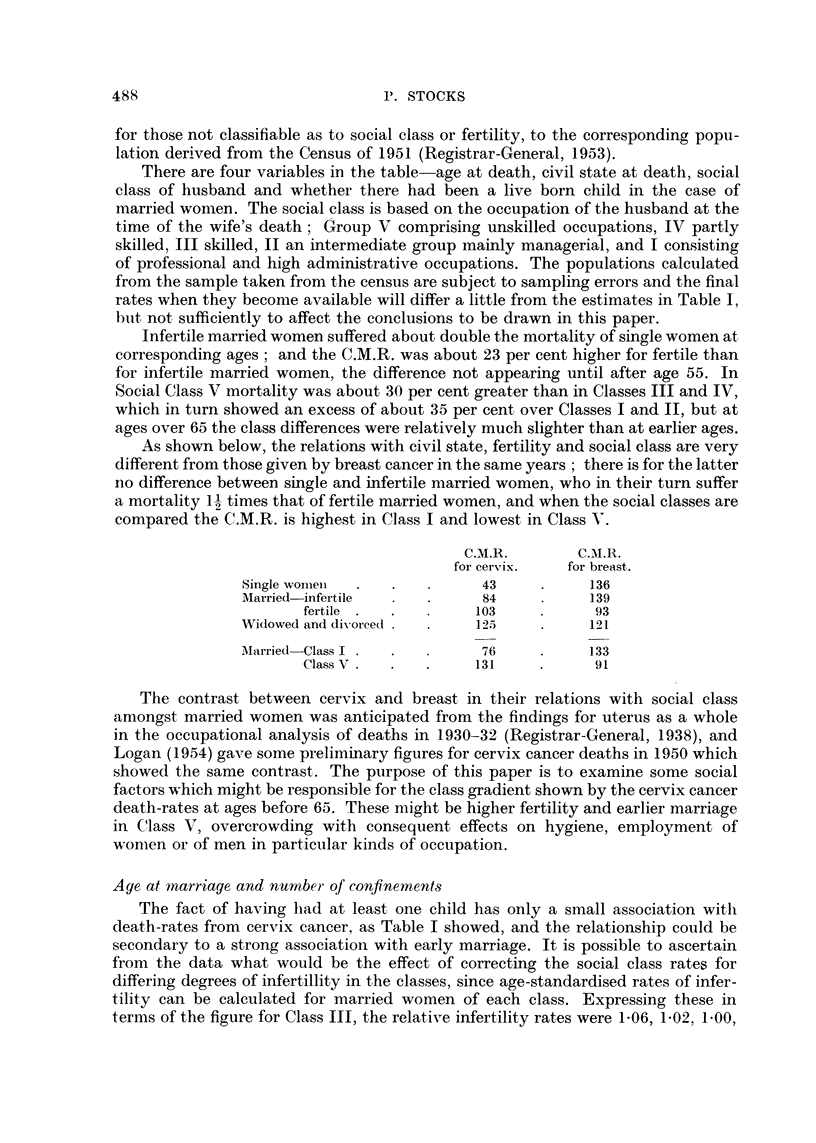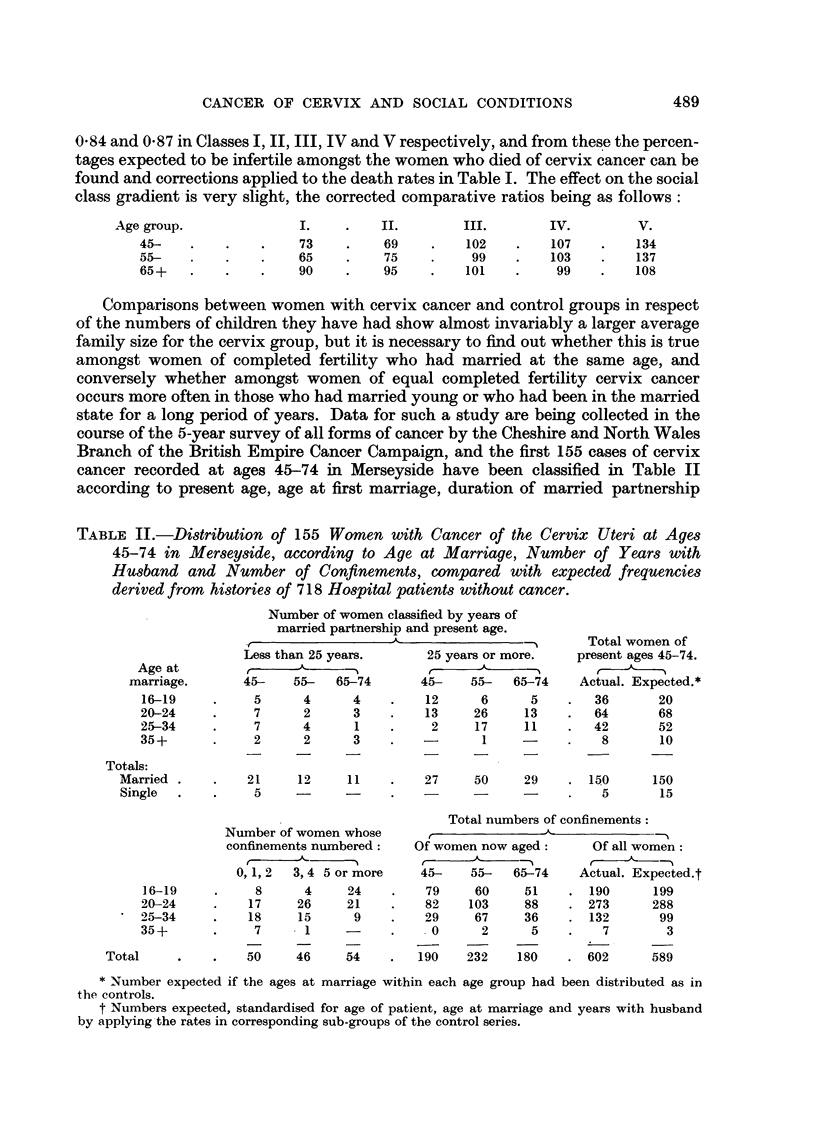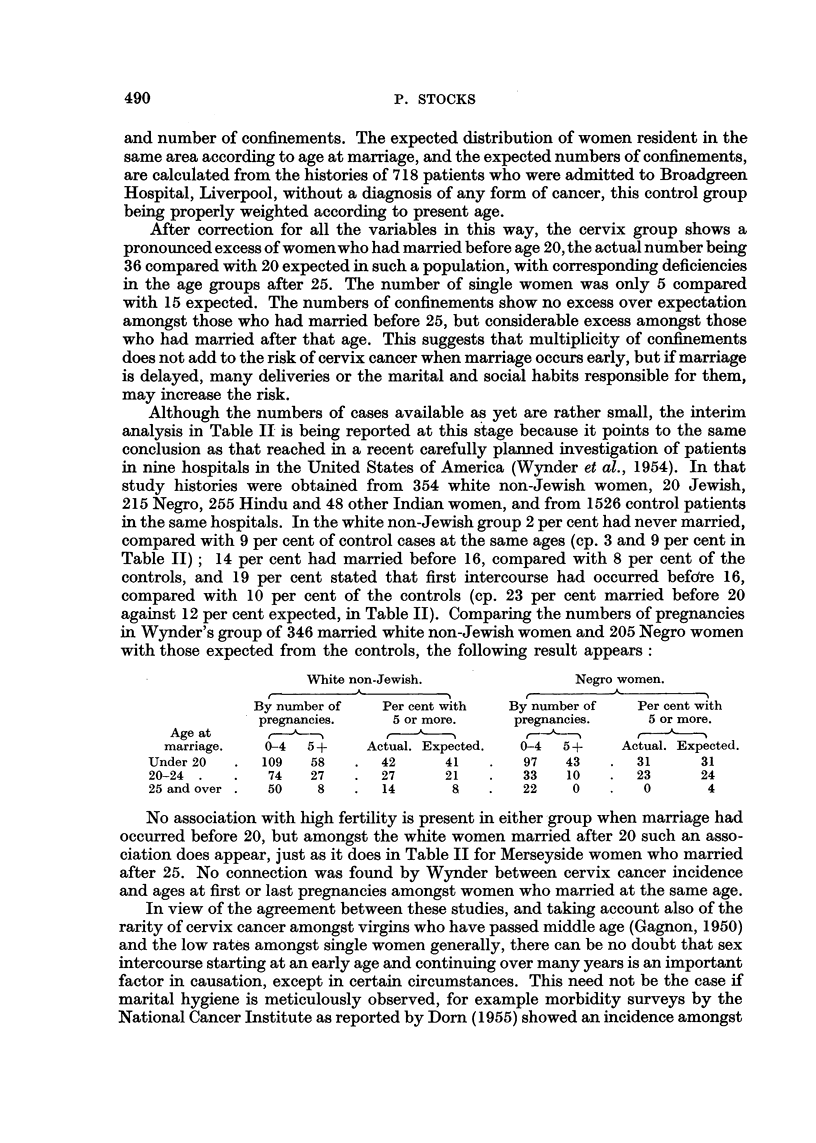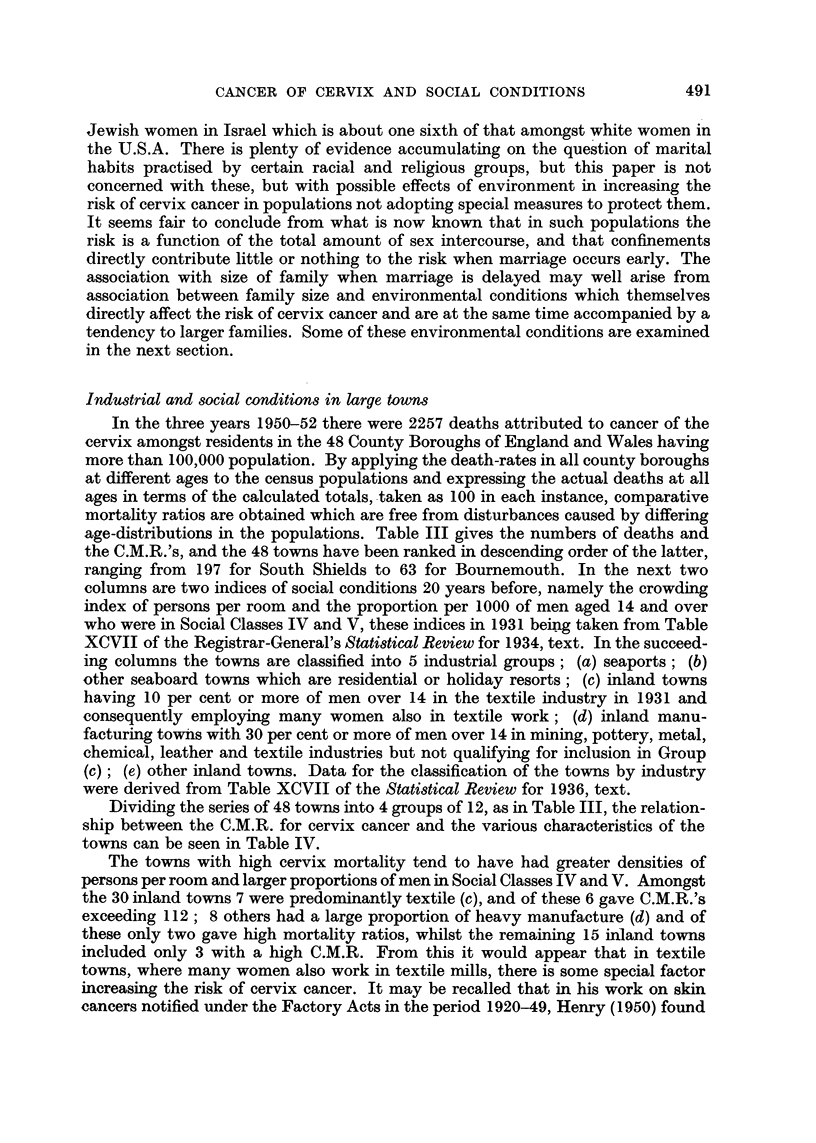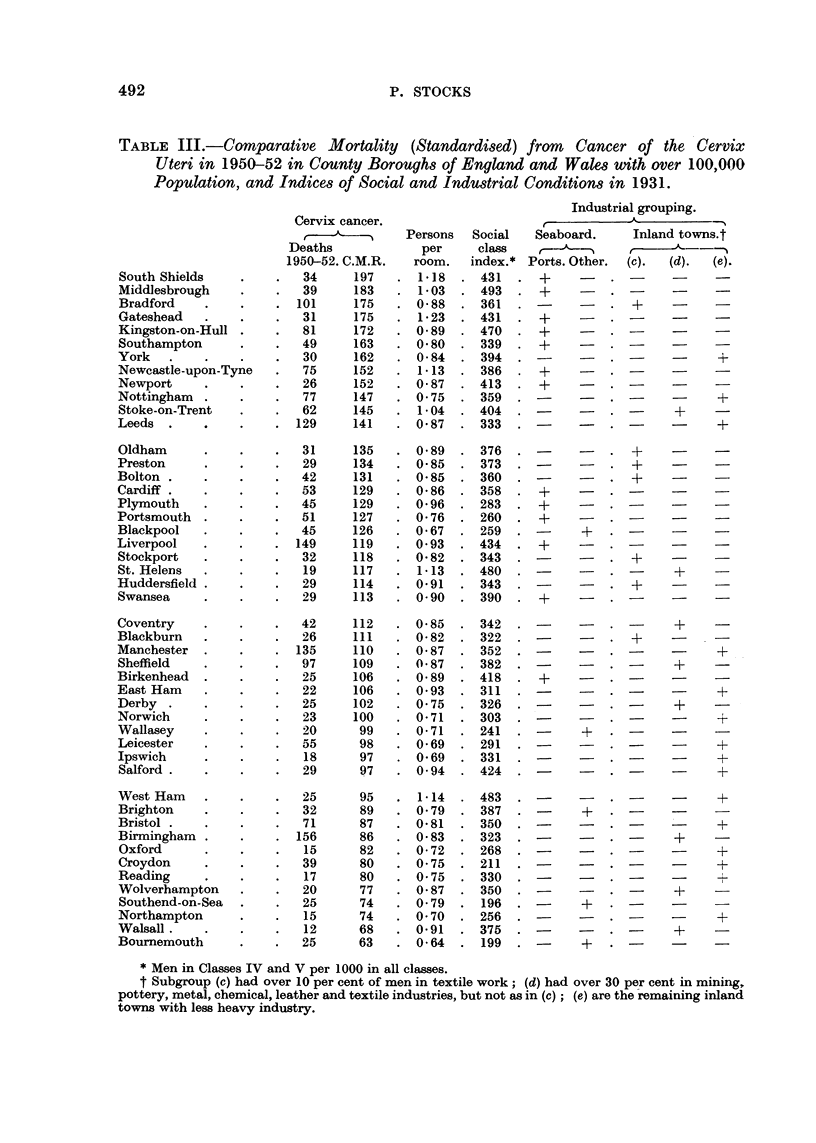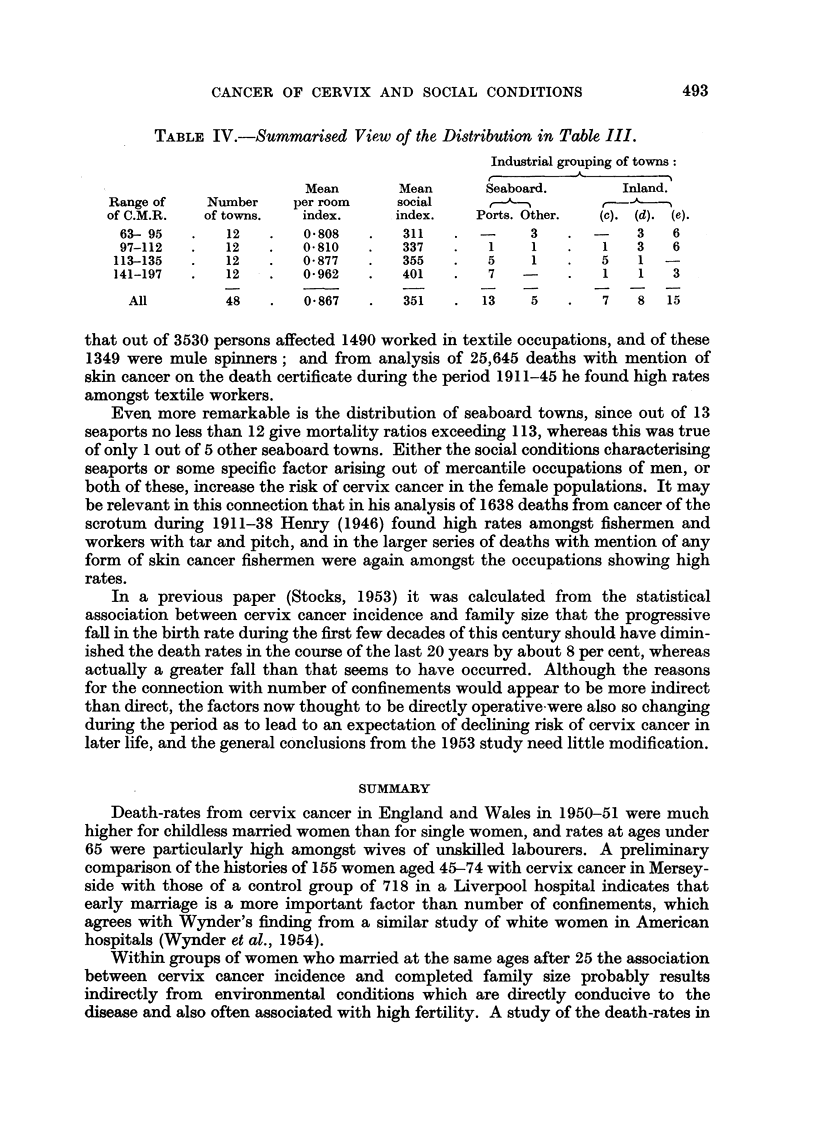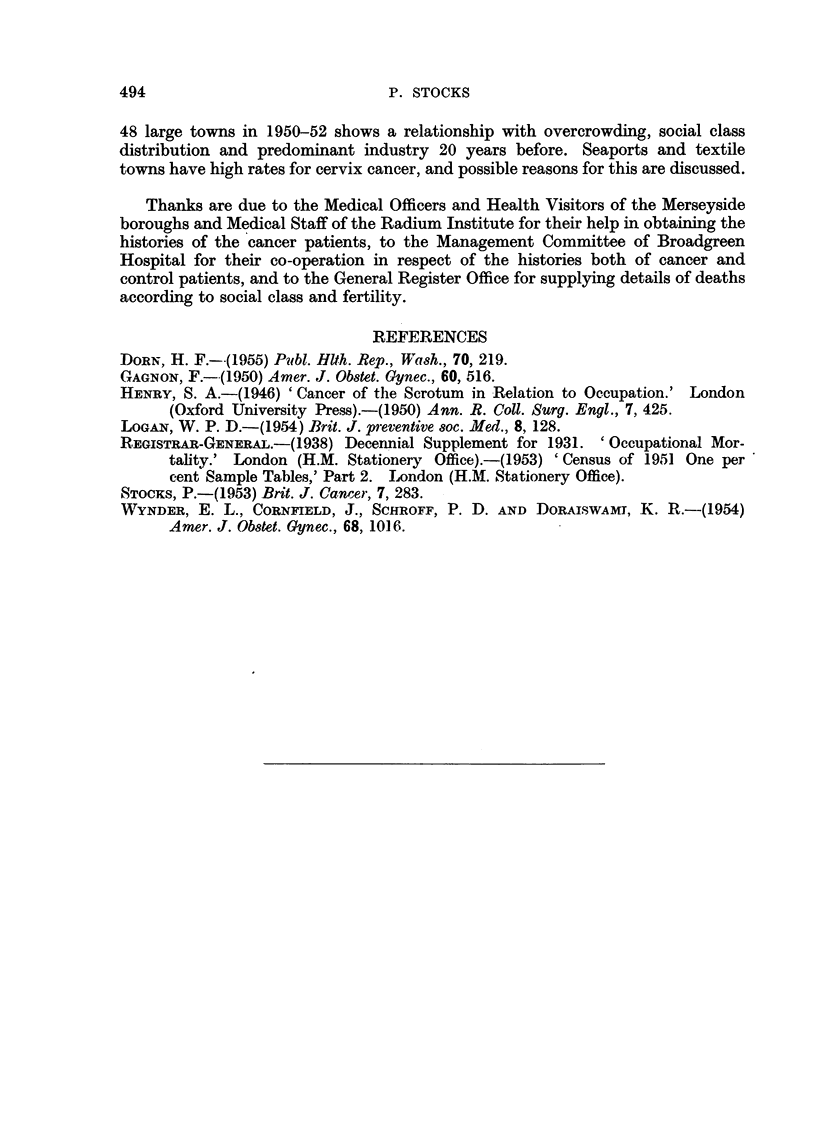# Cancer of the Uterine Cervix and Social Conditions

**DOI:** 10.1038/bjc.1955.51

**Published:** 1955-12

**Authors:** P. Stocks


BRITISH JOURNAL OF CANCER

VOL. IX DECEMBER, 1955 NO. 4

### CANCER OF THE UTERINE CERVIX AND SOCIAL CONDITIONS

P. STOCKS*

*From the Cheshire and North Wales Branch of the British Empire Campaign, Westminster Chambers, St. Werburgh Street, Chester*

Received for publication September 8, 1955

### *Marriage, infertility and social class*

The Registrar-General of England and Wales commenced in 1950 to include amongst the subjects for medical inquiry concerning statements on death certificates cancer of the " uterus " without specification of the part of the uterus in which the neoplasm was present or was thought to have originated. The results of this have been such as to produce meaningful statistics of cervix cancer mortality which were never obtainable up to that time. In order to assist my work for the British Empire Cancer Campaign the General Registrar Office kindly extracted for me the numbers of deaths attributed to this cause during 1950 and 1951, classified according to age, marital condition and, in the case of married women, the social class of the husband and whether or not there had been any children. From these data in respect of 5287 deaths the rates and comparative mortality figures shown in Table I were computed by relating the deaths, after correction

TABLE I.—*Death-rates per Million from Cancer of Cervix Uteri, by Age and Marital Condition, and by Fertility and Social Class of Married Women. Comparative Mortality Ratios in terms of All Married Women taken as* 100. *England and Wales,* 1950–51.

| | Age group. | Single all. | Married. Infertile. | Married. All. | Married. Fertile. | Widowed and divorced (all). |
|---|---|---|---|---|---|---|
| Death rates | 25– | 9 | — | 20 | — | 41 |
| | 35– | 39 | — | 70 | — | 205 |
| | 45– | 69 | 188 | 191 | 192 | 295 |
| | 55– | 136 | 244 | 314 | 325 | 395 |
| | 65 and over | 150 | 306 | 346 | 352 | 423 |
| C.M.R. | All ages | 43 | 84 | 100 | 103 | 125 |

*Married Women by Social Class.*

| | Age group | I. | II. | III. | IV. | V. |
|---|---|---|---|---|---|---|
| Death rates | 25– | 16 | 14 | 19 | 23 | 36 |
| | 35– | 61 | 50 | 75 | 73 | 103 |
| | 45– | 140 | 131 | 194 | 205 | 255 |
| | 55– | 203 | 234 | 310 | 323 | 430 |
| | 65 and over | 310 | 327 | 348 | 345 | 376 |
| C.M.R. | All ages | 76 | 77 | 101 | 104 | 131 |

* Senior Research Fellow, British Empire Cancer Campaign.

for those not classifiable as to social class or fertility, to the corresponding population derived from the Census of 1951 (Registrar-General, 1953).

There are four variables in the table—age at death, civil state at death, social class of husband and whether there had been a live born child in the case of married women. The social class is based on the occupation of the husband at the time of the wife's death ; Group V comprising unskilled occupations, IV partly skilled, III skilled, II an intermediate group mainly managerial, and I consisting of professional and high administrative occupations. The populations calculated from the sample taken from the census are subject to sampling errors and the final rates when they become available will differ a little from the estimates in Table I, but not sufficiently to affect the conclusions to be drawn in this paper.

Infertile married women suffered about double the mortality of single women at corresponding ages ; and the C.M.R. was about 23 per cent higher for fertile than for infertile married women, the difference not appearing until after age 55. In Social Class V mortality was about 30 per cent greater than in Classes III and IV, which in turn showed an excess of about 35 per cent over Classes I and II, but at ages over 65 the class differences were relatively much slighter than at earlier ages.

As shown below, the relations with civil state, fertility and social class are very different from those given by breast cancer in the same years ; there is for the latter no difference between single and infertile married women, who in their turn suffer a mortality $1\frac{1}{2}$ times that of fertile married women, and when the social classes are compared the C.M.R. is highest in Class I and lowest in Class V.

| | C.M.R. for cervix. | C.M.R. for breast. |
|---|---|---|
| Single women | 43 | 136 |
| Married—infertile | 84 | 139 |
| fertile | 103 | 93 |
| Widowed and divorced | 125 | 121 |
| Married—Class I | 76 | 133 |
| Class V | 131 | 91 |

The contrast between cervix and breast in their relations with social class amongst married women was anticipated from the findings for uterus as a whole in the occupational analysis of deaths in 1930–32 (Registrar-General, 1938), and Logan (1954) gave some preliminary figures for cervix cancer deaths in 1950 which showed the same contrast. The purpose of this paper is to examine some social factors which might be responsible for the class gradient shown by the cervix cancer death-rates at ages before 65. These might be higher fertility and earlier marriage in Class V, overcrowding with consequent effects on hygiene, employment of women or of men in particular kinds of occupation.

*Age at marriage and number of confinements*

The fact of having had at least one child has only a small association with death-rates from cervix cancer, as Table I showed, and the relationship could be secondary to a strong association with early marriage. It is possible to ascertain from the data what would be the effect of correcting the social class rates for differing degrees of infertillity in the classes, since age-standardised rates of infertility can be calculated for married women of each class. Expressing these in terms of the figure for Class III, the relative infertility rates were 1·06, 1·02, 1·00,

0·84 and 0·87 in Classes I, II, III, IV and V respectively, and from these the percentages expected to be infertile amongst the women who died of cervix cancer can be found and corrections applied to the death rates in Table I. The effect on the social class gradient is very slight, the corrected comparative ratios being as follows :

| Age group. | I. | II. | III. | IV. | V. |
|---|---|---|---|---|---|
| 45– | 73 | 69 | 102 | 107 | 134 |
| 55– | 65 | 75 | 99 | 103 | 137 |
| 65+ | 90 | 95 | 101 | 99 | 108 |

Comparisons between women with cervix cancer and control groups in respect of the numbers of children they have had show almost invariably a larger average family size for the cervix group, but it is necessary to find out whether this is true amongst women of completed fertility who had married at the same age, and conversely whether amongst women of equal completed fertility cervix cancer occurs more often in those who had married young or who had been in the married state for a long period of years. Data for such a study are being collected in the course of the 5-year survey of all forms of cancer by the Cheshire and North Wales Branch of the British Empire Cancer Campaign, and the first 155 cases of cervix cancer recorded at ages 45–74 in Merseyside have been classified in Table II according to present age, age at first marriage, duration of married partnership

TABLE II.—*Distribution of* 155 *Women with Cancer of the Cervix Uteri at Ages* 45–74 *in Merseyside, according to Age at Marriage, Number of Years with Husband and Number of Confinements, compared with expected frequencies derived from histories of* 718 *Hospital patients without cancer.*

| Age at marriage. | Number of women classified by years of married partnership and present age. | | | | | | Total women of present ages 45–74. | |
|---|---|---|---|---|---|---|---|---|
| | Less than 25 years. | | | 25 years or more. | | | | |
| | 45– | 55– | 65–74 | 45– | 55– | 65–74 | Actual. | Expected.* |
| 16–19 | 5 | 4 | 4 | 12 | 6 | 5 | 36 | 20 |
| 20–24 | 7 | 2 | 3 | 13 | 26 | 13 | 64 | 68 |
| 25–34 | 7 | 4 | 1 | 2 | 17 | 11 | 42 | 52 |
| 35+ | 2 | 2 | 3 | — | 1 | — | 8 | 10 |
| Totals: | | | | | | | | |
| Married | 21 | 12 | 11 | 27 | 50 | 29 | 150 | 150 |
| Single | 5 | — | — | — | — | — | 5 | 15 |

| Age at marriage. | Number of women whose confinements numbered : | | | Total numbers of confinements : Of women now aged : | | | Of all women : | |
|---|---|---|---|---|---|---|---|---|
| | 0, 1, 2 | 3, 4 | 5 or more | 45– | 55– | 65–74 | Actual. | Expected.† |
| 16–19 | 8 | 4 | 24 | 79 | 60 | 51 | 190 | 199 |
| 20–24 | 17 | 26 | 21 | 82 | 103 | 88 | 273 | 288 |
| 25–34 | 18 | 15 | 9 | 29 | 67 | 36 | 132 | 99 |
| 35+ | 7 | 1 | — | 0 | 2 | 5 | 7 | 3 |
| Total | 50 | 46 | 54 | 190 | 232 | 180 | 602 | 589 |

* Number expected if the ages at marriage within each age group had been distributed as in the controls.

† Numbers expected, standardised for age of patient, age at marriage and years with husband by applying the rates in corresponding sub-groups of the control series.

and number of confinements. The expected distribution of women resident in the same area according to age at marriage, and the expected numbers of confinements, are calculated from the histories of 718 patients who were admitted to Broadgreen Hospital, Liverpool, without a diagnosis of any form of cancer, this control group being properly weighted according to present age.

After correction for all the variables in this way, the cervix group shows a pronounced excess of women who had married before age 20, the actual number being 36 compared with 20 expected in such a population, with corresponding deficiencies in the age groups after 25. The number of single women was only 5 compared with 15 expected. The numbers of confinements show no excess over expectation amongst those who had married before 25, but considerable excess amongst those who had married after that age. This suggests that multiplicity of confinements does not add to the risk of cervix cancer when marriage occurs early, but if marriage is delayed, many deliveries or the marital and social habits responsible for them, may increase the risk.

Although the numbers of cases available as yet are rather small, the interim analysis in Table II is being reported at this stage because it points to the same conclusion as that reached in a recent carefully planned investigation of patients in nine hospitals in the United States of America (Wynder *et al.*, 1954). In that study histories were obtained from 354 white non-Jewish women, 20 Jewish, 215 Negro, 255 Hindu and 48 other Indian women, and from 1526 control patients in the same hospitals. In the white non-Jewish group 2 per cent had never married, compared with 9 per cent of control cases at the same ages (cp. 3 and 9 per cent in Table II); 14 per cent had married before 16, compared with 8 per cent of the controls, and 19 per cent stated that first intercourse had occurred before 16, compared with 10 per cent of the controls (cp. 23 per cent married before 20 against 12 per cent expected, in Table II). Comparing the numbers of pregnancies in Wynder's group of 346 married white non-Jewish women and 205 Negro women with those expected from the controls, the following result appears :

| | White non-Jewish. | | | | Negro women. | | | |
|---|---|---|---|---|---|---|---|---|
| | By number of pregnancies. | | Per cent with 5 or more. | | By number of pregnancies. | | Per cent with 5 or more. | |
| Age at marriage. | 0–4 | 5+ | Actual. | Expected. | 0–4 | 5+ | Actual. | Expected. |
| Under 20 | 109 | 58 | 42 | 41 | 97 | 43 | 31 | 31 |
| 20–24 | 74 | 27 | 27 | 21 | 33 | 10 | 23 | 24 |
| 25 and over | 50 | 8 | 14 | 8 | 22 | 0 | 0 | 4 |

No association with high fertility is present in either group when marriage had occurred before 20, but amongst the white women married after 20 such an association does appear, just as it does in Table II for Merseyside women who married after 25. No connection was found by Wynder between cervix cancer incidence and ages at first or last pregnancies amongst women who married at the same age.

In view of the agreement between these studies, and taking account also of the rarity of cervix cancer amongst virgins who have passed middle age (Gagnon, 1950) and the low rates amongst single women generally, there can be no doubt that sex intercourse starting at an early age and continuing over many years is an important factor in causation, except in certain circumstances. This need not be the case if marital hygiene is meticulously observed, for example morbidity surveys by the National Cancer Institute as reported by Dorn (1955) showed an incidence amongst

Jewish women in Israel which is about one sixth of that amongst white women in the U.S.A. There is plenty of evidence accumulating on the question of marital habits practised by certain racial and religious groups, but this paper is not concerned with these, but with possible effects of environment in increasing the risk of cervix cancer in populations not adopting special measures to protect them. It seems fair to conclude from what is now known that in such populations the risk is a function of the total amount of sex intercourse, and that confinements directly contribute little or nothing to the risk when marriage occurs early. The association with size of family when marriage is delayed may well arise from association between family size and environmental conditions which themselves directly affect the risk of cervix cancer and are at the same time accompanied by a tendency to larger families. Some of these environmental conditions are examined in the next section.

*Industrial and social conditions in large towns*

In the three years 1950–52 there were 2257 deaths attributed to cancer of the cervix amongst residents in the 48 County Boroughs of England and Wales having more than 100,000 population. By applying the death-rates in all county boroughs at different ages to the census populations and expressing the actual deaths at all ages in terms of the calculated totals, taken as 100 in each instance, comparative mortality ratios are obtained which are free from disturbances caused by differing age-distributions in the populations. Table III gives the numbers of deaths and the C.M.R.'s, and the 48 towns have been ranked in descending order of the latter, ranging from 197 for South Shields to 63 for Bournemouth. In the next two columns are two indices of social conditions 20 years before, namely the crowding index of persons per room and the proportion per 1000 of men aged 14 and over who were in Social Classes IV and V, these indices in 1931 being taken from Table XCVII of the Registrar-General's *Statistical Review* for 1934, text. In the succeeding columns the towns are classified into 5 industrial groups ; (*a*) seaports ; (*b*) other seaboard towns which are residential or holiday resorts ; (*c*) inland towns having 10 per cent or more of men over 14 in the textile industry in 1931 and consequently employing many women also in textile work ; (*d*) inland manufacturing towns with 30 per cent or more of men over 14 in mining, pottery, metal, chemical, leather and textile industries but not qualifying for inclusion in Group (*c*) ; (*e*) other inland towns. Data for the classification of the towns by industry were derived from Table XCVII of the *Statistical Review* for 1936, text.

Dividing the series of 48 towns into 4 groups of 12, as in Table III, the relationship between the C.M.R. for cervix cancer and the various characteristics of the towns can be seen in Table IV.

The towns with high cervix mortality tend to have had greater densities of persons per room and larger proportions of men in Social Classes IV and V. Amongst the 30 inland towns 7 were predominantly textile (*c*), and of these 6 gave C.M.R.'s exceeding 112 ; 8 others had a large proportion of heavy manufacture (*d*) and of these only two gave high mortality ratios, whilst the remaining 15 inland towns included only 3 with a high C.M.R. From this it would appear that in textile towns, where many women also work in textile mills, there is some special factor increasing the risk of cervix cancer. It may be recalled that in his work on skin cancers notified under the Factory Acts in the period 1920–49, Henry (1950) found

TABLE III.—*Comparative Mortality (Standardised) from Cancer of the Cervix Uteri in* 1950–52 *in County Boroughs of England and Wales with over* 100,000 *Population, and Indices of Social and Industrial Conditions in* 1931.

| | Cervix cancer. | | | | Industrial grouping. | | | | |
|---|---|---|---|---|---|---|---|---|---|
| | | | | | Seaboard. | | Inland towns.† | | |
| | Deaths 1950–52. | C.M.R. | Persons per room. | Social class index.* | Ports. | Other. | (*c*). | (*d*). | (*e*). |
| South Shields | 34 | 197 | 1·18 | 431 | + | — | — | — | — |
| Middlesbrough | 39 | 183 | 1·03 | 493 | + | — | — | — | — |
| Bradford | 101 | 175 | 0·88 | 361 | — | — | + | — | — |
| Gateshead | 31 | 175 | 1·23 | 431 | + | — | — | — | — |
| Kingston-on-Hull | 81 | 172 | 0·89 | 470 | + | — | — | — | — |
| Southampton | 49 | 163 | 0·80 | 339 | + | — | — | — | — |
| York | 30 | 162 | 0·84 | 394 | — | — | — | — | + |
| Newcastle-upon-Tyne | 75 | 152 | 1·13 | 386 | + | — | — | — | — |
| Newport | 26 | 152 | 0·87 | 413 | + | — | — | — | — |
| Nottingham | 77 | 147 | 0·75 | 359 | — | — | — | — | + |
| Stoke-on-Trent | 62 | 145 | 1·04 | 404 | — | — | — | + | — |
| Leeds | 129 | 141 | 0·87 | 333 | — | — | — | — | + |
| Oldham | 31 | 135 | 0·89 | 376 | — | — | + | — | — |
| Preston | 29 | 134 | 0·85 | 373 | — | — | + | — | — |
| Bolton | 42 | 131 | 0·85 | 360 | — | — | + | — | — |
| Cardiff | 53 | 129 | 0·86 | 358 | + | — | — | — | — |
| Plymouth | 45 | 129 | 0·96 | 283 | + | — | — | — | — |
| Portsmouth | 51 | 127 | 0·76 | 260 | + | — | — | — | — |
| Blackpool | 45 | 126 | 0·67 | 259 | — | + | — | — | — |
| Liverpool | 149 | 119 | 0·93 | 434 | + | — | — | — | — |
| Stockport | 32 | 118 | 0·82 | 343 | — | — | + | — | — |
| St. Helens | 19 | 117 | 1·13 | 480 | — | — | — | + | — |
| Huddersfield | 29 | 114 | 0·91 | 343 | — | — | + | — | — |
| Swansea | 29 | 113 | 0·90 | 390 | + | — | — | — | — |
| Coventry | 42 | 112 | 0·85 | 342 | — | — | — | + | — |
| Blackburn | 26 | 111 | 0·82 | 322 | — | — | + | — | — |
| Manchester | 135 | 110 | 0·87 | 352 | — | — | — | — | + |
| Sheffield | 97 | 109 | 0·87 | 382 | — | — | — | + | — |
| Birkenhead | 25 | 106 | 0·89 | 418 | + | — | — | — | — |
| East Ham | 22 | 106 | 0·93 | 311 | — | — | — | — | + |
| Derby | 25 | 102 | 0·75 | 326 | — | — | — | + | — |
| Norwich | 23 | 100 | 0·71 | 303 | — | — | — | — | + |
| Wallasey | 20 | 99 | 0·71 | 241 | — | + | — | — | — |
| Leicester | 55 | 98 | 0·69 | 291 | — | — | — | — | + |
| Ipswich | 18 | 97 | 0·69 | 331 | — | — | — | — | + |
| Salford | 29 | 97 | 0·94 | 424 | — | — | — | — | + |
| West Ham | 25 | 95 | 1·14 | 483 | — | — | — | — | + |
| Brighton | 32 | 89 | 0·79 | 387 | — | + | — | — | — |
| Bristol | 71 | 87 | 0·81 | 350 | — | — | — | — | + |
| Birmingham | 156 | 86 | 0·83 | 323 | — | — | — | + | — |
| Oxford | 15 | 82 | 0·72 | 268 | — | — | — | — | + |
| Croydon | 39 | 80 | 0·75 | 211 | — | — | — | — | + |
| Reading | 17 | 80 | 0·75 | 330 | — | — | — | — | + |
| Wolverhampton | 20 | 77 | 0·87 | 350 | — | — | — | + | — |
| Southend-on-Sea | 25 | 74 | 0·79 | 196 | — | + | — | — | — |
| Northampton | 15 | 74 | 0·70 | 256 | — | — | — | — | + |
| Walsall | 12 | 68 | 0·91 | 375 | — | — | — | + | — |
| Bournemouth | 25 | 63 | 0·64 | 199 | — | + | — | — | — |

* Men in Classes IV and V per 1000 in all classes.

† Subgroup (*c*) had over 10 per cent of men in textile work ; (*d*) had over 30 per cent in mining, pottery, metal, chemical, leather and textile industries, but not as in (*c*) ; (*e*) are the remaining inland towns with less heavy industry.

TABLE IV.—*Summarised View of the Distribution in Table III.*

| Range of of C.M.R. | Number of towns. | Mean per room index. | Mean social index. | Industrial grouping of towns: Seaboard. Ports. | Other. | Inland. (c). | (d). | (e). |
|---|---|---|---|---|---|---|---|---|
| 63– 95 | 12 | 0·808 | 311 | — | 3 | — | 3 | 6 |
| 97–112 | 12 | 0·810 | 337 | 1 | 1 | 1 | 3 | 6 |
| 113–135 | 12 | 0·877 | 355 | 5 | 1 | 5 | 1 | — |
| 141–197 | 12 | 0·962 | 401 | 7 | — | 1 | 1 | 3 |
| All | 48 | 0·867 | 351 | 13 | 5 | 7 | 8 | 15 |

that out of 3530 persons affected 1490 worked in textile occupations, and of these 1349 were mule spinners; and from analysis of 25,645 deaths with mention of skin cancer on the death certificate during the period 1911–45 he found high rates amongst textile workers.

Even more remarkable is the distribution of seaboard towns, since out of 13 seaports no less than 12 give mortality ratios exceeding 113, whereas this was true of only 1 out of 5 other seaboard towns. Either the social conditions characterising seaports or some specific factor arising out of mercantile occupations of men, or both of these, increase the risk of cervix cancer in the female populations. It may be relevant in this connection that in his analysis of 1638 deaths from cancer of the scrotum during 1911–38 Henry (1946) found high rates amongst fishermen and workers with tar and pitch, and in the larger series of deaths with mention of any form of skin cancer fishermen were again amongst the occupations showing high rates.

In a previous paper (Stocks, 1953) it was calculated from the statistical association between cervix cancer incidence and family size that the progressive fall in the birth rate during the first few decades of this century should have diminished the death rates in the course of the last 20 years by about 8 per cent, whereas actually a greater fall than that seems to have occurred. Although the reasons for the connection with number of confinements would appear to be more indirect than direct, the factors now thought to be directly operative were also so changing during the period as to lead to an expectation of declining risk of cervix cancer in later life, and the general conclusions from the 1953 study need little modification.

### SUMMARY

Death-rates from cervix cancer in England and Wales in 1950–51 were much higher for childless married women than for single women, and rates at ages under 65 were particularly high amongst wives of unskilled labourers. A preliminary comparison of the histories of 155 women aged 45–74 with cervix cancer in Merseyside with those of a control group of 718 in a Liverpool hospital indicates that early marriage is a more important factor than number of confinements, which agrees with Wynder's finding from a similar study of white women in American hospitals (Wynder *et al.*, 1954).

Within groups of women who married at the same ages after 25 the association between cervix cancer incidence and completed family size probably results indirectly from environmental conditions which are directly conducive to the disease and also often associated with high fertility. A study of the death-rates in

48 large towns in 1950–52 shows a relationship with overcrowding, social class distribution and predominant industry 20 years before. Seaports and textile towns have high rates for cervix cancer, and possible reasons for this are discussed.

Thanks are due to the Medical Officers and Health Visitors of the Merseyside boroughs and Medical Staff of the Radium Institute for their help in obtaining the histories of the cancer patients, to the Management Committee of Broadgreen Hospital for their co-operation in respect of the histories both of cancer and control patients, and to the General Register Office for supplying details of deaths according to social class and fertility.

### REFERENCES

Dorn, H. F.—(1955) *Publ. Hlth. Rep., Wash.*, **70**, 219.
Gagnon, F.—(1950) *Amer. J. Obstet. Gynec.*, **60**, 516.
Henry, S. A.—(1946) 'Cancer of the Scrotum in Relation to Occupation.' London (Oxford University Press).—(1950) *Ann. R. Coll. Surg. Engl.*, **7**, 425.
Logan, W. P. D.—(1954) *Brit. J. preventive soc. Med.*, **8**, 128.
Registrar-General.—(1938) Decennial Supplement for 1931. 'Occupational Mortality.' London (H.M. Stationery Office).—(1953) 'Census of 1951 One per cent Sample Tables,' Part 2. London (H.M. Stationery Office).
Stocks, P.—(1953) *Brit. J. Cancer*, **7**, 283.
Wynder, E. L., Cornfield, J., Schroff, P. D. and Doraiswami, K. R.—(1954) *Amer. J. Obstet. Gynec.*, **68**, 1016.